# Genetic Factors Explain Half of the Individual Susceptibility to Chronic Bronchitis, Airflow Obstruction and COPD Regardless of the Spirometric Definition: A Nationwide Twin Study

**DOI:** 10.1007/s00408-025-00825-3

**Published:** 2025-06-27

**Authors:** Howraman Meteran, Simon Francis Thomsen, Jacob Hjelmborg, Martin R. Miller, Kaare Christensen, Torben Sigsgaard, Vibeke Backer

**Affiliations:** 1 Department of Respiratory Medicine, Copenhagen University Hospital - Hvidovre, Hvidovre, Denmark; 2 Department of Public Health, Environment, Occupation and Health, Aarhus University, Aarhus C, Denmark; 3 Department Respiratory Medicine, Zealand University Hospital – Roskilde/Næstved, Næstved, Denmark; 4 Department of Dermatology, Venereology and Wound Healing Centre, Copenhagen University Hospital – Bispebjerg, Copenhagen, Denmark; 5 Department of Biomedical Sciences, University of Copenhagen, Copenhagen, Denmark; 6 Department of Epidemiology and Biostatistics, University of Southern Denmark, Odense, Denmark; 7 The Danish Twin Registry, University of Southern Denmark, Odense, Denmark; 8 Institute of Occupational and Environmental Medicine, University of Birmingham, Birmingham, UK; 9 Department of Otorhinolaryngology, Head and Neck Surgery and Audiology, Copenhagen University Hospital - Rigshospitalet, Copenhagen, Denmark

**Keywords:** Lung function, COPD, Chronic bronchitis, Heritability, Twin

## Abstract

**Background:**

Smoking is a major risk factor for lung function decline and chronic obstructive pulmonary disease (COPD), but the individual susceptibility to these traits cannot be explained solely by environmental risk factors.

**Aim:**

To estimate the relative contribution of genetic and environmental factors to lung function, chronic bronchitis and COPD.

**Methods:**

12,449 twins aged 40–80 years participated in a nationwide survey using the Danish Twin Registry, which included a questionnaire, clinical examination and spirometry. Clinical COPD was defined by respiratory symptoms plus airflow obstruction. Biometric models of genetic and environmental latent factors were used to estimate the heritability after adjusting for sex, age, and height.

**Results:**

Mean (SD) age of the study population was 58.4 (9.6) years and mean BMI (kg/m^2^) was 26.6 (4.4); 20% were current smokers and 52% were females. The heritability of FEV_1_, FVC and FEV_1_/FVC was 64% (60–67%), 61% (57–65%), and 50% (46–55%), respectively. Genetic factors explained 48% (24–72%) and 47% (16–78%), respectively, of the individual susceptibility to chronic bronchitis and clinical COPD.

**Conclusion:**

Genetic factors explain at least half of the variation in lung function and around half of the individual susceptibility to chronic bronchitis and clinical COPD, respectively, when adjusted for sex, age, height.

**Supplementary Information:**

The online version contains supplementary material available at 10.1007/s00408-025-00825-3.

## Introduction

Chronic obstructive pulmonary disease (COPD) is a major non-communicable chronic disease [1] characterized by chronic inflammation and destruction of the lung parenchyma, which leads to chronic airflow obstruction [2]. COPD patients experience symptoms such a shortness of breath, cough, and mucus production which worsen during an exacerbation [3]. Symptoms and chronic airflow obstruction are both required for the diagnosis of COPD [4]. Chronic bronchitis is a key component of COPD and is defined as “chronic cough and sputum from the airways for at least three months in each of two successive years, as long as there are no other causes of chronic cough” [5].

Smoking is a major risk factor for both lung function decline [6], chronic bronchitis [7] and COPD [8]. However, more recent studies have shown that less than half of all smokers develop COPD [9, 43] and other risk factors such as air pollution from biomass fuels and occupation [10], socio-economic status [11] and diet [12, 13] have been identified, but they are only accountable for 20% of the COPD cases [14]. Thus, it has been suggested that the individual susceptibility could be due to genetic factors. The most well-known genetic risk factor is α−1-antitrypsin deficiency present in 1–2% of the COPD population [15].

Previous studies have shown a familial aggregation in COPD [16, 17] and the genetic influence on lung function [18, 19], chronic bronchitis [20, 21] and COPD [22] has been estimated. However, the relative genetic and environmental influence on these traits has never been estimated concomitantly and, in particularly, in a population-based sample. Furthermore, the heritability of airflow obstruction defined according to ATS/ERS [4] recommendations has to our knowledge never been reported.

Thus, the aims of this study were to estimate the genetic and environmental contributions to the individual variation in i) lung function measured by FEV_1_, (*forced expiratory volume in first second*) FVC (*forced vital capacity),* FEV_1_/FVC-ratio, ii) airflow obstruction defined according to GOLD guidelines [3] and ATS/ERS guidelines [4], and iii) chronic bronchitis and COPD.

## Methods

### Design

Data were collected from the Danish nationwide survey, the Infrastructure Initiative which took place in 2008–2011. The study sample for the current study is based on two cohorts of Middle Age Danish Twins from the Danish Twin Registry, consisting of 2,402 individuals born 1931–1952 (MADT), and a further 10,281 individuals born 1931–1969 (MIDT). The data collection was based on a questionnaire filled out at home and a clinical examination, which took place in the three largest cities in Denmark: Copenhagen, Aarhus, and Odense. The participation rate was 62% for the MADT cohort and about 40% for the MIDT cohort. The study was approved by The Danish National Committee on Biomedical Research Ethics.

### Study Population

Questionnaire data were available for 12,449 twin individuals and lung function data were available for 11,219 individuals (90%). The heritability of lung function was estimated using the whole population.

Prior to the estimation of the genetic and environmental contribution to the various definitions of airflow obstruction, chronic bronchitis and clinical COPD, participants with self-reported asthma were excluded (*n* = 991) leaving 11,458 individuals eligible for inclusion for these analyses. Among these, lung function data were available for 10,329 individuals.

### Questionnaire and Spirometry Data

Questionnaire data were used to determine zygosity by asking four questions of similarity and mistaken identity, which is a method with an overall misclassification of 4% compared with genetic marker analysis [23]. *Chronic bronchitis* was defined if the participant gave an affirmative response to the question ‘Have you experienced chronic cough and sputum for at least three months per year in the past 2 years or more?’ The participants were classified with ‘respiratory symptoms’ if they had an affirmative response to one of the following questions ‘Do you experience shortness of breath at rest or during exercise?’, ‘Do you have a cough?’, ‘Do you have nocturnal awakening due to shortness of breath or chest tightness?’.

Airflow obstruction was defined as either FR-AO (*fixed ratio-airflow obstruction*): FEV1/FVC-ratio < 0.70, LLN2.5-AO (*lower limit of normal-airflow obstruction*) using a *z*-score of < − 1.96. Clinical COPD was defined as the presence of respiratory symptoms and an FEV_1_/FVC-ratio below the LLN. We conducted sensitivity analyses using a *z*-score of − 1.645 corresponding to the lowest 5% of the population to define lower limit of normal for both airflow obstruction (LLN5-AO) and clinical COPD (LLN5-COPD).

#### Statistical Analysis

Biometric models of genetic and environmental latent factors were fitted to the raw data using the polygenic ADCE model of quantitative genetics, described by Neale and Cardon [24]. In this approach it is assumed that the individual variation in the susceptibility to a given trait is a product of both genetic and environmental factors and thus, the total variance for a phenotypic trait can be expressed as:

$$V_{{{\text{Total}}}} = V_{{\text{A}}} + V_{{\text{D}}} + V_{{\text{C}}} + V_{{\text{E}}}$$

Another main assumption is that monozygotic (MZ) twins share 100% of their inherited genes and dizygotic (DZ) twins have on average half of their genes identical by descent, so if genetic factors influence the trait under consideration MZ twins should be more similar than DZ twins. The genetic variance can be decomposed into *additive genetic effect*s, A (loci contributing additively to the risk of disease) and *non-additive genetic effects*, D (interacting alleles: *dominant* (from same locus) or *epistasis* (from different loci)). The environmental factors can be decomposed into *common, shared environmental factors*, C and into *unique environmental factors*, E (which also includes measurement error).

Based on these assumptions, the expected covariance in MZ and DZ twin can be derived:

$${\text{CoV}}_{{{\text{MZ}}}} = V_{{\text{A}}} + V_{{\text{D}}} + V_{{\text{C}}}$$

$${\text{Co}}V_{{{\text{DZ}}}} = 0.{5}V_{{\text{A}}} + 0.{25}V_{{\text{D}}} + V_{{\text{C}}}$$

Dichotomous outcomes were assumed to be observations of a latent liability and the bivariate liability-threshold model was used implementing the above ADCE components and sex, age, height as covariates. We note that no measurement of time of occurrence of the (dichotomous) outcome was available for adjusting for effects due to not having complete information at follow-up, i.e., censoring. For most human traits it is reasonable to assume that all four sources of variance (A, D, C, and E) act simultaneously. However, only the effect of either C or D can be estimated in the same model [25]. Thus, the choice between either an ACE or ADE model is given by the model with the lowest AIC (*Akaike Information Criterion*), which provides a mean for selection between various models [26]. The significance of the contribution of the individual components to the variance in disease liability was determined by a likelihood-ratio test for the difference between the full ACE/ADE models and subsequently fitted nested models (AE and CE models). The results were adjusted for the effects of sex, age, and height.

Analyses for descriptive statistics were conducted using the statistical package Stata 14.0 (StataCorp LLC, Texas, USA) and variance component analyses were conducted using the METS package available in the R statistical software (version 3.1.1) [27, 28].

## Results

Table 1 shows the baseline characteristics of the population. The mean age of the study participants was 58.4 years; 52% were females and the mean BMI (kg/m2) was 26.6 ± 4.4. All three results remained unchanged after exclusion of asthmatic individuals. The prevalence of current, former and never smokers in the total population was 20%, 38% and 42%, respectively. A lower prevalence of current smokers was observed in the FR-AO group compared with LLN2.5-AO After exclusion of asthmatic individuals, the prevalence of chronic bronchitis and COPD was 3.7% and 2.5%, respectively.

**Table 1:** Baseline characteristics in the total sample

Patient characteristics	Total sample (*n* = 12,449)	After exclusion of self-reported asthma			
		Total sample (*n* = 11,458)	Fixed ratio only (*n* = 1278)	Lower limit of normal 2.5 (*z*-score < − 1.96) (*n* = 576)	Lower limit of normal 5 (*z*-score < − 1.645) (*n* = 946)
Sex (male), No. (%)	5,962 (48%)	5,849 (48%)	673 (53%)	265 (46%)	407 (43%)
Age, mean (SD)	58.4 (9.6)	58.4 (9.6)	63.3 (8.7)	60.0 (9.3)	59.4 (9.5)
*Body Mass Index (BMI)*					
Overall, mean (SD)	*26.6 (4.4)*	*26.6 (4.4)*	*26.3 (4.2)*	*25.4 (4.5)*	*25.6 (4.4)*
Underweight, *n* (%)	96 (1)	85 (1)	9 (1)	13 (2)	18 (2)
Normal weight, *n* (%)	4,655 (38)	4,320 (38)	514 (40)	279 (49)	444 (47)
Overweight, *n* (%)	5,283 (43)	4,868 (43)	542 (43)	196 (34)	344 (36)
Obese, *n* (%)	2,324 (19)	2,097 (18)	210 (16)	86 (15)	137 (15)
*Smoking, n (%)*					
Current smoking	2,454 (20)	2,273 (20)	389 (31)	289 (50)	420 (45)
Former smoking	4,690 (38)	4,262 (37)	514 (40)	194 (34)	334 (36)
Never smoking	5,221 (42)	4,842 (43)	369 (29)	90 (16)	186 (20)
Pack-years, mean (SD)	19.2 (18.0)	19.2 (18.0)	24.5 (19.7)	30.3 (21.5)	27.6 (20.8)
*Z-score for spirometric in dex, mean (SD)*					
FEV_1_	− 0.30 (1.11)	− 0.25 (1.08)	− 0.84 (0.94)	− 1.57 (1.10)	− 1.90 (1.10)
FVC	− 0.08 (1.01)	− 0.05 (1.00)	0.01 (1.08)	− 0.22 (1.27)	− 0.39 (1.35)
FEV_1_/FVC	− 0.42 (1.01)	− 0.37 (0.98)	− 1.43 (0.31)	− 2.27 (0.58)	− 2.58 (0.55)

The number of individuals in the FR-AO-only group (*n* = 1278) is based on distinction from LLN defined by a *z*-score < − 1.96

The percentages are for available data and not the total number

**Fig. 1:**
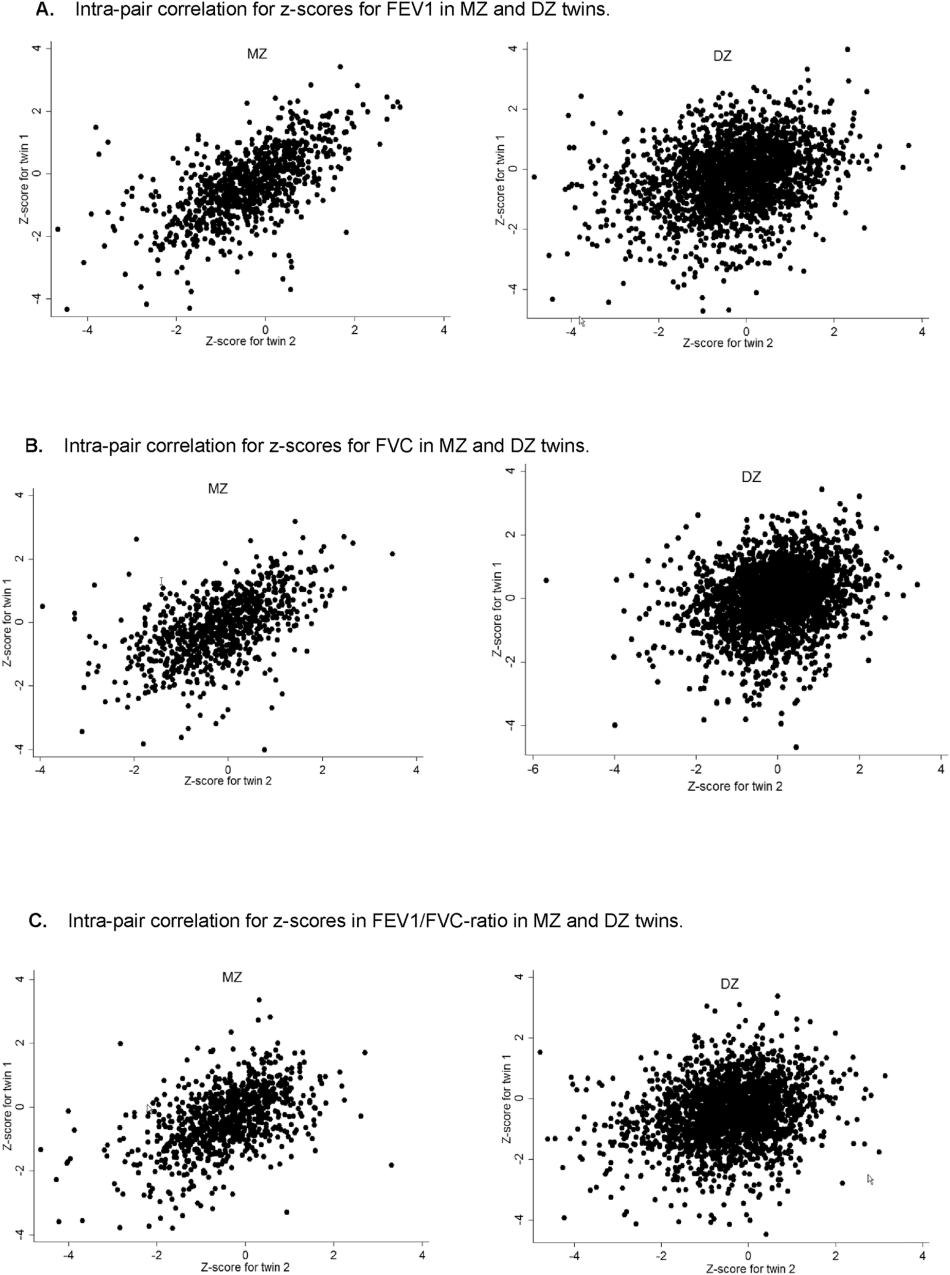
**a** Intra-pair correlation for *z*-scores for FEV1 in MZ and DZ twins. **b** Intra-pair correlation for *z*-scores for FVC in MZ and DZ twins. **c** Intra-pair correlation for *z*-scores in FEV1/FVC-ratio in MZ and DZ twins

Figure 1A–C illustrate the intra-pair correlation *z*-scores for FEV_1_, FVC, and FEV_1_/FVC-ratio in MZ and DZ twins. The graphical inspection indicates a stronger correlation in MZ twins for all three indices.

**Fig. 2:**
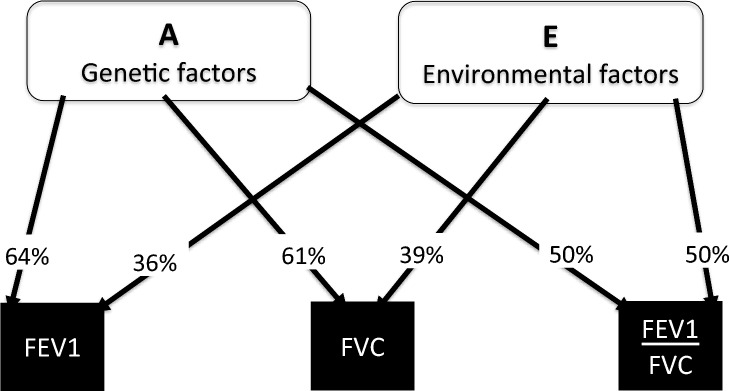
The relative contribution of genetic and environmental factors on the variation in spirometric indices. additive genetic factors, E: unique environmental factors

The variance component analyses of lung function indices are presented in Table 2 and illustrated on Fig. 2. Comparison between the saturated model and the nested models showed that the AE model was the preferred for both FEV_1_, FVC and FEV_1_/FVC-ratio. The relative contribution of genetic and unique environmental factors to the variation in the indices was 64% (60–67%) and 36% (33–40%) respectively for FEV_1_, 61% (57–65%) and 39% (35–43%) respectively for FVC, and 50% (46–55%) and 50% (45–54%) respectively for the FEV_1_/FVC-ratio.

**Table 2:** The relative contribution of genetic and environmental factors on the variation in spirometric indices, A (additive genes), D (non-additive genes), C (shared environmental factors), and E (unique environmental factors)

Spirometric index	Variance components			Fit statistics			Correlation (95% CI)	
	A	C/D	E	AIC	d*f*	*P*-value	MZ	DZ
***FEV1***				*5185.8*	*26*		*0.63 (0.59–0.67)*	*0.29 (0.23–0.34)*
ADE	0.47 (0.27–0.68)	0.17 (− 0.04–0.38)	0.35 (0.32–0.39)	5174.4			0.64 (0.61–0.68)	0.28 (0.28–0.33)
** AE**	**0.64 (0.60–0.67)**		**0.36 (0.33–0.40)**	**5174.9**	**1**	**0.11**	**0.64 (0.60–0.67)**	**0.32 (0.30–0.34)**
CE		0.42 (0.39–0.46)	0.58 (0.54–0.61)	5298.8			0.42 (0.39–0.46)	0.42 (0.39–0.46)
***FVC***				*6355.6*	*26*		*0.61 (0.56–0.65)*	*0.26 (0.21–0.32)*
ADE	0.41 (0.20–0.62)	0.21 (0.01–0.42)	0.38 (0.34–0.42)	6352.3			0.62 (0.58–0.66)	0.26 (0.21–0.31)
** AE**	**0.61 (0.57–0.65)**		**0.39 (0.35–0.43)**	**6353.9**	**1**	**0.06**	**0.61 (0.57–0.65)**	**0.30 (0.28–0.32)**
CE		0.40 (0.36–0.44)	0.60 (0.56–0.64)	6466.4			0.40 (0.36–0.44)	0.40 (0.36–0.44)
***FEV1/FVC***				− *9765.4*	*26*		*0.49 (0.44–0.54)*	*0.24 (0.19–0.30)*
ADE	0.43 (0.23–0.63)	0.08 (− 0.13–0.29)	0.49 (0.44–0.54)	− 9773.6			0.51 (0.46–0.56)	0.24 (0.19–0.28)
** AE**	**0.50 (0.46–0.55)**		**0.50 (0.45–0.54)**	− **9775.1**	**1**	**0.49**	**0.50 (0.46–0.55)**	**0.25 (0.23–0.28)**
CE	0.34 (0.30–0.38)		0.66 (0.62–0.70)	− 9721.1			0.34 (0.30–0.38)	0.34 (0.30–0.38)

AIC (Akaike Information Criteria), df (Degrees of freedom), MZ and DZ correlations for the saturated model are printed in italic. For each model the preferred model based on parsimony and fit statistics is highlighted in bold and shows the estimates for each of the included components. The ‘df’ reflects the estimated parameters in the model.

The *p*-value is derived from a likelihood-ratio test and reflects the difference between the full model and nested model and should be non-significant to use the nested model. Only *p*-value for the most parsimonious model is given

Table S1 shows the variance component analyses for the spirometric indices stratified by smoking status (never, former and current smoking). The genetic contribution to the individual variation in both FEV_1_, FVC and FEV_1_/FVC-ratio was increased in former and current smokers compared with never smokers, which could suggest that smoking enhances the effect of genetic factors on the variation in lung function, however, this needs further investigation.

**Fig. 3:**
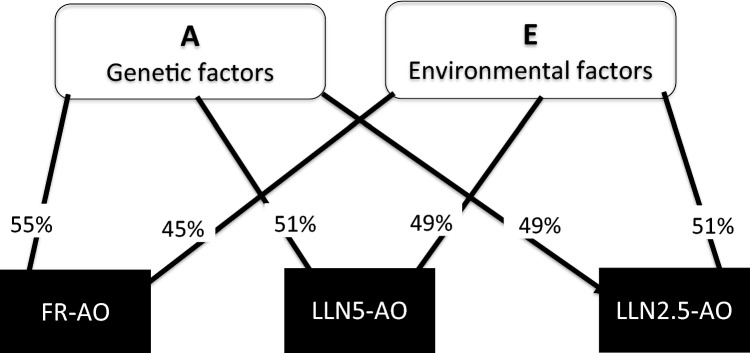
The relative contribution of genetic and environmental factors on the individual susceptibility to airflow obstruction according to different spirometric criteria. A: additive genetic factors, E: unique environmental factors, FR-AO: Fixed ratio obstruction, LLN5-AO (lower limit of normal, *z*-score < 1.64) for FEV1/FVC-ratio, LLN5-AO (lower limit of normal, *z*-score < − 1.64) for FEV1/FVC-ratio, and LLN2.5-AO (lower limit of normal, *z*-score < − 1–96 for FEV1/FVC-ratio)

Table 3 shows that the preferred models for all three different spirometric definitions of airflow obstruction were the AE models. Tetrachoric correlations (measure of similarity of twin pairs) in MZ twins were stronger than in DZ same sex twins for all three definitions: FR-AO; 57% (44–69%) vs. 22% (25–36%), LLN5-AO; 59% (41–72%) vs. 9% (− 10–34%), and LLN2.5-AO; 55% (24–73%) vs. 17% (− 8–40%), respectively. Additive genetic and unique environmental factors explained the individual susceptibility to FR-AO; 55% (43–66%) and 45% (34–57%), LLN5-AO; 51% (35–68%) and 49% (32–65%), LLN2.5-AO; 49% (26–72%) and 51% (28–71%), respectively as shown on Fig. 3.

**Table 3:** The relative contribution of genetic and environmental factors on the individual susceptibility to airflow obstruction according to different spirometric criteria, A (additive genes), D (non-additive genes), C (shared environmental factors), and E (unique environmental factors)

Airflow obstruction (N)	Variance components			Fit statistics			Tetrachoric correlations (95% CI)	
	A	C/D	E	AIC	d*f*	*P*-value	MZ	DZ
***FR-AO*** ***(1,854)***							*0.57 (0.44–0.69)*	*0.22 (0.25–0.36)*
ADE	0.31 (− 0.20–0.82)	0.26 (− 0.30–0.82)	0.43 (0.30–0.55)	5401.3	7		0.57 (0.44–0.69)	0.22 (0.09–0.34)
** AE**	**0.55 (0.43–0.66)**		**0.45 (0.34–0.57)**	**5400.2**	**1**	**0.36**	**0.55 (0.42–0.65)**	**0.27 (0.21–0.33)**
CE		0.36 (0.27–0.46)	0.64 (0.54–0.73)	5413.3			0.36 (0.27–0.45)	0.36 (0.27–0.45)
***LLN5-AO*** ***(946)***							*0.59 (0.41–0.72)*	*0.09 (*− *0.10–0.27)*
ADE	0.00 (0.00–0.00)	0.58 (0.42–0.74)	0.42 (0.26–0.58)	3660.5			0.58 (0.39–0.72)	0.14 (0.10–0.18)
** AE**	**0.51 (0.35–0.68)**		**0.49 (0.32–0.65)**	**3662.3**	**1**	** > 0.05**	**0.51 (0.33–0.66)**	**0.26 (0.17–0.34)**
CE		0.31 (0.18–0.44)	0.69 (0.56–0.82)	3673.2			0.31 (0.18–0.43)	0.31 (0.18–0.43)
***LLN2.5-AO (576)***							*0.54 (0.26–0.73)*	*0.17 (*− *0.08–0.40)*
ADE	0.13 (− 0.87–1.13)	0.40 (− 0.68–1.48)	0.46 (0.23–0.70)	2515.9	7		0.54 (0.26–0.73)	0.17 (− 0.08–0.40)
** AE**	**0.49 (0.26–0.72)**		**0.51 (0.28–0.74)**	**2514.2**	**1**	**0.46**	**0.49 (0.23–0.69)**	**0.25 (0.13–0.36)**
CE		0.32 (0.14–0.49)	0.68 (0.51–0.86)	2518.2			0.32 (0.13–0.48)	0.32 (0.13–0.48)

Tetrachoric correlations in MZ and DZ twins for the various definitions of airflow obstruction for the unsaturated model are printed in italic

For each model the preferred model based on parsimony and fit statistics is highlighted in bold and shows the estimates for each of the included components. All individuals with self-reported physician-diagnosed asthma excluded

FR-AO: fixed ratio-airflow obstruction; FEV1/FVC < 0.7. LLN5-AO: lower limit of normal-airflow obstruction; FEV1/FVC < LLN (*z*-score of − 1.645), and LLN2.5-AO: lower limit of normal, FEV1/FVC < LLN (*z*-score of − 1.96), respectively. The *p*-value reflects the difference between the full model and nested model and should be non-significant to use the nested model. Only *p*-values for the most parsimonious model is given

**Fig. 4:**
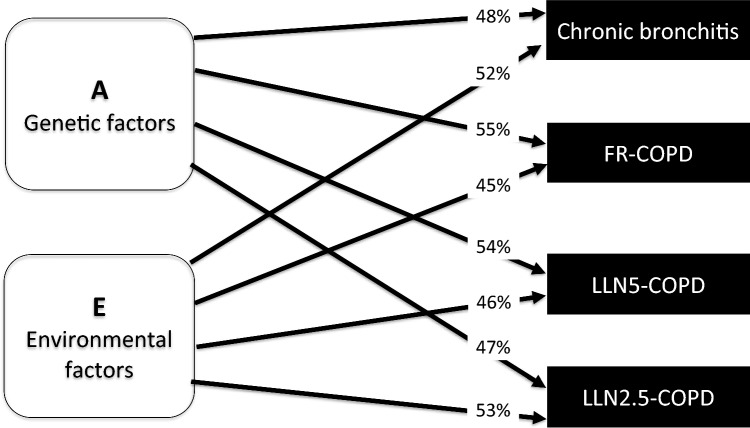
The relative contribution of genetic and environmental factors on the individual susceptibility to FEV1, FVC, chronic bronchitis and clinical COPD according to different spirometric definitions. A: Additive genetic factors, E: Unique environmental factors. Chronic bronchitis defined as chronic cough with sputum for at least three months in two consecutive years. LLN2.5-COPD (*z*-score < − 1.64), LLN5-COPD (*z*-score < − 1.96), and FR-COPD (fixed ratio COPD)

Table 4 shows that the tetrachoric correlations for MZ twins are higher than DZ same sex twins for chronic bronchitis; 45% (12–69%) vs. 30% (3–53%) and LLN2.5-COPD; 52% (15–76%) vs. 12% (− 28–38%), respectively. Both chronic bronchitis and LLN2.5-COPD were best described by a model which included additive genetic factors and unique environmental factors with a relative contribution for chronic bronchitis; 48% (24–72%) and 52% (28–76%), respectively, and for LLN2.5-COPD; 47% (16–78%) and 53% (22–84%), respectively. Further, we conducted two sensitivity analyses using both FR and LLN5 (*z*-score of − 1.645) to diagnose COPD and estimated the genetic and environmental contribution: FR-COPD; 55% (38–72%) and 45% (28–62%) and for LLN5-COPD; 54% (32–77%) and 46% (23–68%), respectively (Table 4 and Fig. 4).

**Table 4:** The relative contribution of genetic and environmental factors on the individual susceptibility to chronic bronchitis and COPD according to different spirometric criteria, A (additive genes), D (non-additive genes), C (shared environmental factors), and E (unique environmental factors)

Clinical outcome (*N*)	Variance components			Fit statistics			Tetrachoric correlations (95% CI)	
	A	C/D	E	AIC	d*f*	*P*-value	MZ	DZ
Chronic bronchitis* **(424)**							*0.45 (0.12–0.69)*	*0.30 (0.03–0.53)*
ACE	0.29 (− 0.48–1.06)	0.16 (− 0.43–0.74)	0.55 (0.27–0.84)	1829.6	7		0.45 (0.27–0.84)	0.30 (0.03–0.53)
** AE**	**0.48 (0.24–0.72)**		**0.52 (0.28–0.76)**	**1827.2**	**1**	**0.61**	**0.48 (0.24–0.72)**	**0.52 (0.28–0.76)**
CE		0.36 (0.17–0.56)	0.64 (0.44–0.83)	1827.9			0.36 (0.16–0.54)	0.36 (0.16–0.54)
**LLN2.5-COPD** **(289)**							*0.52 (0.15–0.76)*	*0.12 (*− *0.28–0.48)*
ADE	0.00 (0.00–0.00)	0.21 (0.01–0.42)	0.38 (0.34–0.42)	1466.2	7		0.51 (0.21–0.82)	0.13 (0.05–0.20)
** AE**	**0.47 (0.16–0.78)**		**0.53 (0.22–0.84)**	**1464.6**	**1**	**0.50**	**0.47 (0.11–0.72)**	**0.23 (0.08–0.38)**
CE		0.31 (0.07–0.56)	0.69 (0.44–0.93)	1466.6			0.31 (0.05–0.54)	0.31 (0.05–0.54)
**LLN5-COPD** **(421)**							*0.60 (0.35–0.77)*	*0.08 (*− *0.23–0.77)*
ADE	0.00 (0.00–0.00)	0.59 (0.38–0.81)	0.41 (0.19–0.77)	1993.4	7		0.59 (0.34–0.77)	0.15 (0.09–0.20)
** AE**	**0.54 (0.32–0.77)**		**0.46 (0.23–0.68)**	**1993.0**	**1**	**0.21**	**0.54 (0.28–0.73)**	**0.27 (0.16–0.38)**
CE		0.36 (0.18–0.55)	0.64 (0.45–0.82)	1998.4			0.36 (0.18–0.55)	0.36 (0.18–0.55)
FR-COPD* **(724)**							*0.51 (0.28–0.69)*	*0.33 (0.14–0.50)*
ACE	0.36 (− 0.19–0.91)	0.15 (− 0.26–0.57)	0.49 (0.28–0.70)	2851.1	7		0.51 (0.28–0.69)	0.33 (0.14–0.50)
** AE**	**0.55 (0.38–0.72)**		**0.45 (0.28–0.62)**	**2849.6**	**1**	**1.00**	**0.55 (0.38–0.72)**	**0.45 (0.28–0.62)**
CE		0.40 (0.26–0.54)	0.60 (0.46–0.74)	2850.6			0.40 (0.25–0.53)	0.40 (0.25–0.53)

Tetrachoric correlations for MZ and DZ twins from the unsaturated model are printed italic. For each model the preferred model based on parsimony and fit statistics is highlighted in bold and shows the estimates for each of the included components

*Chronic bronchitis defined as chronic cough with sputum for at least three months in two consecutive years. LLN2.5-COPD (*z*-score < − 1.64), LLN5-COPD (*z*-score < − 1.96), and FR-COPD (fixed ratio COPD). All individuals with self-reported physician-diagnosed asthma excluded. The *p*-value reflects the difference between the full model and nested model and should be non-significant to use the nested model. Only *p*-values for the most parsimonious model is given

## Discussion

This study shows that genetic factors explain more than half of individual susceptibility to the lung function indices FEV_1_, FVC, FEV_1_/FVC-ratio and half of the variance in airflow obstruction, chronic bronchitis and COPD. Only a few studies have attempted to quantify the genetic effect on lung function [29] [30] [19] [42]. In a small twin study (414 pairs), Redline et al. estimated the intra-pair correlations for FEV_1_, FVC, FEV_1_/FVC-ratio to be 72%, 76% and 55%, respectively [29]. In a Swedish study of 230 twin pairs, McClearn et al. found that genetic factors account for 48% and 67% of the variation in vital capacity (VC) and FEV_1_, respectively [30]. In another small study of 74 twin pairs, Ghio et al. were not able to find any overwhelming evidence of heritability of lung function [31]. It should be underlined that these early studies differ from ours in both sample size, selection of participant and methodology. In a more recent, but also small study including 379 twin pairs, Tian et al. found similar heritability estimates for FEV1 (55%), but no significant heritability for FVC [42]. In line with our findings a more recent Danish twin study by Ingebrigtsen et al. found that more than half of the individual variation in FEV_1_ (61%) and FVC (55%) was explained by genetic factors[19]. However, no estimate of the genetic influence on FEV_1_/FVC-ratio was provided.

To our knowledge, this is the first study to investigate and compare the heritability of multiple spirometric criteria for airflow obstruction in parallel. We found that additive genetic factors explained half of the variation in susceptibility to all three spirometric definitions of airflow obstruction. Further, sensitivity analyses in which asthmatic individuals were included did not change the overall findings. A limited number of studies reporting the heritability of chronic bronchitis have been performed[21] [20]. Hallberg et al. estimated a 40% heritability of chronic bronchitis [21] and we have in another, registry-based study estimated the heritability to be 55% in women [20], whereas the familial aggregation in men seemed to be ascribable to environmental factors. Other studies have found that chronic bronchitis in first-degree relatives is a stronger predictor of disease in the unaffected individual than is smoking [32]. Asthma is a common cause of chronic bronchitis inflammation [33] and we cannot be certain whether we had a mixture of both asthma and COPD patients in our previous study [20], whereas the present heritability estimates of chronic bronchitis are based on a population with a larger age span and exclusion of self-reported physician-diagnosed asthma.

Further, additive genetic factors explained 47% of the individual susceptibility to COPD in this population-based twin study and thus, moderately lower than the estimate (63%) found in another Danish twin study [22]. This could be due to the study design as they used hospital discharge diagnosis, which therefore, represents a group of COPD patients with exacerbations and severe disease. In a study by McCloskey and colleagues, siblings of patient with severe COPD had a five-time increased risk of COPD when compared with controls [17] and in a another study, Patel et al. found that both airway thickening and emphysema show independent familial aggregation [16].

To date, a number of relevant genetic determinants have been identified. The first and most well-known genetic risk factor is severe α−1-antitrypsin deficiency (AAT), encoded by the SERPINA1 gene and accounts for 1–2% of the COPD population [15]. In the recent years it has been shown that augmentation treatment for AAT preserves the lung parenchyma in patients with emphysema [34]. In the last decade a number of genome-wide association studies (GWAS) have been conducted [35, 36]. Some of the first studies revealed significant associations between the CHRNA3/CHRNA5/IREB2 region on chromosome 15q25 [35], TGFβR3 and an increased risk of COPD [36].

Studies suggest that specific genetic factors are associated with various COPD-phenotypes including lung function and emphysema. In a large population-based GWAS based on cohorts from the UK Biobank and SpiroMeta Consortium, approximately 300 significant loci for spirometric indices have been identified [37]. A large study using chest CT scans to characterize the distribution and severity of emphysema have found genome-wide associations in the SNRPF and PPT2 regions [38]. In another GWAS comprised data from COPDGene, ECLIPSE, and GenKOLS two previously reported genome-wide significant associations (HHIP, chromosome 15q25) and three novel loci (SOWAHB, TRAPPC9, and KIAA1462) were found [39].

Since the clinical COPD diagnosis is based on impaired lung function defined by thresholds, a number of shared loci have been reported. In the recent years, it has become evident that COPD is not only caused by rapid lung function decline, but also abnormal growth and development [40]. A study by Moll et al. found that polygenic risk scores was associated with several clinical COPD entities such as emphysema subtypes and lung function growth pattern [44]. Thus, further studies aiming to determine the genetic influence on lung function trajectories are warranted.

This study has a number of strengths. It is the first study to estimate the relative contribution of genetic and environmental factors on the variation in FEV_1_, FVC and FEV1/FVC-ratio, airflow obstruction defined according to different spirometric criteria, chronic bronchitis and clinical COPD in the same population. Further, to our knowledge this is the largest study including unselected twin pairs and the nationwide design of the study increase the generalizability, although extrapolation of findings from one single study to the general population can be questioned. Further, some possible limitations should be mentioned. We did not perform post-bronchodilator spirometry, which is required in diagnosing airflow obstruction according to GOLD, but the misclassification is reduced as we excluded all individuals with self-reported physician-diagnosed asthma. Since we used questionnaire data to diagnose chronic bronchitis, we cannot reject possible recall bias. However, chronic bronchitis was diagnosed according to current guidelines [5] and the correlation between self-reported chronic bronchitis and a clinical confirmation of sputum production has been shown [41]. 

In conclusion, this is the largest twin study to estimate the relative genetic and environmental contribution on the variance in lung function, airflow obstruction, chronic bronchitis and clinical COPD in an unselected study population. Further, this is the first study to estimate the heritability in airflow obstruction and clinical COPD defined by both fixed ratio and two cut-off values for lower limit of normal. Genetic factors explain about 60% of the individual variation in lung function and around 50% of the individual susceptibility to chronic bronchitis and COPD. Therefore, physicians should be aware of patients with a family history of chronic bronchitis and COPD regardless of smoking status.

*What is the key question?* What is the relative genetic and environmental contribution on the variation in lung function and risk of chronic bronchitis and COPD defined according to various spirometric criteria?

*What is the bottom line?* More than half of the individual variation in lung function and risk of COPD regardless of the spirometric criteria is explained by genetic factors.

*Why read on?* This study it the first of its kind to quantify the genetic contribution to both lung function and COPD using different spirometric criteria.

## Supplementary Information

Below is the link to the electronic supplementary material.

Supplementary file1 (DOCX 43 kb)

## Data Availability

No datasets were generated or analysed during the current study.
