# Supplementary material for: Genetic Factors Explain Half of the Individual Susceptibility to Chronic Bronchitis, Airflow Obstruction and COPD Regardless of the Spirometric Definition: A Nationwide Twin Study
Source: Lung. 2025 Jun 27;203(1):70. doi: 10.1007/s00408-025-00825-3 (PMC12204911; doi:10.1007/s00408-025-00825-3)
Supplement: Supplementary file 1 — Supplementary file1 (DOCX 43 kb) [file 408_2025_825_MOESM1_ESM.docx]

**Supplemental material**

**Table S1.** The relative contribution of genetic and environmental factors on the variation in spirometric indices, A (additive genes), D (non-additive genes), C (shared environmental factors), and E (unique environmental factors) stratified by smoking status.

| **Spirometric index** | **Variance components** | | | **Correlation (95% CI)** | |
| --- | --- | --- | --- | --- | --- |
|  | **A** | **C/D** | **E** | **MZ** | **DZ** |
| ***FEV1*** |  |  |  |  |  |
| ***Smoking*** |  |  |  |  |  |
| Never | 0.63 (0.57-0.70) |  | 0.37 (0.30-0.43) | 0.63 (0.56-0.69) | 0.32 (0.28-0.35) |
| Former | 0.71 (0.63-0.78) |  | 0.29 (0.22-0.37) | 0.71 (0.62-0.77) | 0.35 (0.31-0.39) |
| Current | 0.74 (0.65-0.83) |  | 0.26 (0.17-0.35) | 0.74 (0.63-0.82) | 0.37 (0.32-0.41) |
|  |  |  |  |  |  |
| ***FVC*** |  |  |  |  |  |
| ***Smoking*** |  |  |  |  |  |
| Never | 0.62 (0.56-0.69) |  | 0.38 (0.31-0.44) | 0.62 (0.56-0.68) | 0.31 (0.28-0.34) |
| Former | 0.70 (0.63-0.78) |  | 0.30 (0.22-0.37) | 0.70 (0.62-0.77) | 0.35 (0.31-0.39) |
| Current | 0.67 (0.56-0.78) |  | 0.33 (0.22-0.44) | 0.67 (0.55-0.77) | 0.34 (0.28-0.39) |
|  |  |  |  |  |  |
| ***FEV1/FVC*** |  |  |  |  |  |
| ***Smoking*** |  |  |  |  |  |
| Never | 0.44 (0.34-0.53) |  | 0.56 (0.47-0.66) | 0.44 (0.34-0.53) | 0.24 (0.17-0.27) |
| Former | 0.46 (0.34-0.57) |  | 0.54 (0.43-0.66) | 0.46 (0.34-0.57) | 0.23 (0.17-0.29) |
| Current | 0.60 (0.47-0.73) |  | 0.40 (0.62-0.70) | 0.34 (0.30-0.38) | 0.34 (0.30-0.38) |
| The given estimates are from the most parsimonious nested model from a full ACE/ADE model for each stratum (never, former and current smoking) for FEV_1,_ FVC, FEV_1_FVC-ratio. | | | | | |

| **Table S2. The four questions used for Zygosity Assessment in the Danish Twin Registry.** |
| --- |
| 1. Do you and your twin look….   - Like two ordinary siblings?  - Like two peas in a pod?*  - Not very much alike?   1. In school is/was it difficult for your teachers and friends to tell you apart? 2. Is/Was it difficult for your teachers and friends to tell you apart? 3. In childhood, did you and your twin have both the same eye color and the same hair color? |
| *The Danish phrase translates to “*like two drops of water*”. |

| **Tables S3. Questions on smoking habits.** |
| --- |
| 1. Do you smoke now?   - Yes, more than one per day  - Yes, but less than one per day  - No  2. Have you previously smoked?  - Yes, more than one per day  - Yes, but less than one per day  - No  3. When did you stop smoking? (year)  4. Do you inhale now (or when you did smoke?  5. How much do (or did) you smoke in average per day?  6. How many years have you been smoking regularly? |
